# Preparation of a Ceramic Matrix Composite Made of Hydroxyapatite Nanoparticles and Polylactic Acid by Consolidation of Composite Granules

**DOI:** 10.3390/nano10061060

**Published:** 2020-05-30

**Authors:** Elzbieta Pietrzykowska, Barbara Romelczyk-Baishya, Jacek Wojnarowicz, Marina Sokolova, Karol Szlazak, Wojciech Swieszkowski, Janis Locs, Witold Lojkowski

**Affiliations:** 1 Institute of High Pressure Physics, Polish Academy of Science, Sokolowska 29/37, 01-142 Warsaw, Poland; j.wojnarowicz@labnano.pl (J.W.); w.lojkowski@labnano.pl (W.L.); 2 Faculty of Materials Science and Engineering, Warsaw University of Technology, Woloska 141, 02-507 Warsaw, Poland; barbara.romelczyk-baishya.dokt@pw.edu.pl (B.R.-B.); karolszlazak@wp.pl (K.S.); wojciech.swieszkowski@pw.edu.pl (W.S.); 3 Rudolfs Cimdins Riga Biomaterials Innovations and Development Centre of RTU, Institute of General Chemical Engineering, Faculty of Materials Science and Applied Chemistry, Riga Technical University, Pulka Street 3, LV-1007 Riga, Latvia; marina.sokolova@rtu.lv (M.S.); janis.locs@rtu.lv (J.L.)

**Keywords:** hybrid, biodegradable, composite, hydroxyapatite, polylactid acid

## Abstract

Composites made of a biodegradable polymer, e.g., polylactic acid (PLA) and hydroxyapatite nanoparticles (HAP NPs) are promising orthopedic materials. There is a particular need for biodegradable hybrid nanocomposites with strong mechanical properties. However, obtaining such composites is challenging, since nanoparticles tend to agglomerate, and it is difficult to achieve good bonding between the hydrophilic ceramic and the hydrophobic polymer. This paper describes a two-step technology for obtaining a ceramic matrix composite. The first step is the preparation of composite granules. The granules are obtained by infiltration of porous granules of HAP NPs with PLA through high-pressure infiltration. The homogeneous ceramic-polymer granules are 80 μm in diameter, and the composite granules are 80 wt% HAP NPs. The second step is consolidation of the granules using high pressure. This is performed in three variants: Uniaxial pressing with the pressure of up to 1000 MPa at room temperature, warm isostatic compaction (75 MPa at 155 °C), and a combination of the two methods. The combined methods result in the highest densification (99%) and strongest mechanical properties; the compressive strength is 374 MPa. The structure of the ceramic matrix composite is homogeneous. Good adhesion between the inorganic and the organic component is observable using scanning electron microscopy.

## 1. Introduction

The orthopedic market experiences a continuous interest in bioresorbable materials, such as phosphates (based on calcium phosphate and calcium carbonate) and polymers. The former group includes hydroxyapatite (HAP) and beta-tricalcium phosphate (β-TCP), which are biocompatible materials whose chemical composition is very similar to the natural apatite found in bone tissue. The calcium phosphate ceramic can improve the bioactivity, osteoconductivity, and resorbability of composite biomaterials [1,2,3]. The market is interested in bioresorbable materials with high mechanical strength, and many studies have been conducted that seek to increase their mechanical properties. The calcium phosphate ceramic is used for cementite, coatings, three-dimensional (3D) printed scaffolds and drug delivery. Due to its brittleness, it cannot be used for implants that must withstand high loads and require high toughness; thus, its applications are limited. The strength limitations concern the present polymer-ceramic materials available on the market [4,5,6].

The most commonly used bioresorbable polymers belong to the group of polyesters that include polycaprolactone (PCL), polylactic acid (PLA) and their copolymers. These are biodegradable materials with degradation times from a few months to several years. They are used for orthopedic implants, such as sutures, bone plates, screws, and scaffolds, as well as aortic valves. In general, orthopedic implants must have specific biological properties (biocompatibility, osseointegration, non-toxicity), but also importantly, they must have mechanical properties that closely match those of the tissue they are replacing [7,8,9,10]. Adequate strength is needed to ensure the transfer of stresses, to provide a base for bone growth and to stimulate the body, in order to regenerate and promote the growth of osteoblasts. For these reasons, an important material parameter for such solutions is Young’s modulus (E). It is assumed that E should be about 12 GPa. Tschakaloff et al. found through in vivo tests using rabbits, based on a histopathologic analysis and computer tomography, that the application of copolymers did not exert a significant impact on the healing of bones. In turn, they observed a big decrease in the molecular weight of the implant made of copolymers over six weeks. They state in the summary of the outcome of the experiment that copolymers can be used if short-term fixing is required, i.e., the short-term strength of the material is required [11].

PLA and its copolymers are biocompatible and biodegradable materials, whose degradation times can be controlled through their structure and molecular weight. They are made of natural ingredients, and one of their advantages is that they can be processed easily, e.g., by the injection method. Their mechanical properties are as follows: Young’s modulus = 2.7 GPa, tensile strength = 50 MPa, and degradation time up to two years [12,13]. However, when compared to bone tissue, these mechanical properties are insufficient. For this reason, they are allowed by the U.S. Food and Drug Administration to be used in orthopedics only when high stiffness of the material is not required. Besides, this polymer is not osteoinductive, and its degradation products may cause local acidification in the body [13,14]. Both industry and research have recently seen such activities as a development of the PLA matrix composites with bioactive phosphate ceramics (HAP, β-TCP). The ceramic content of such solutions is up to 10% [15,16,17]. The addition of ceramics should lead to an increase in E, improve biological properties, and neutralize acidification in the tissue surrounding the implant. However, the present solutions are insufficient regarding degradation characteristics and mechanical properties, and the implants cannot sustain heavy loads [18,19,20,21]. 

Composites offer opportunities to design new materials by combining different material properties, e.g., combining the high compressive strength of ceramics with the high toughness of bioresorbable polymers [22]. By controlling the proportion and structure of such materials, we can improve their strength and Young’s modulus and give them properties similar to natural bones. The use of hard bioactive ceramics improve composite stiffness and osteoinduction. There are several such solutions known in the literature [23,24,25]; however, they are not bioresorbable materials. 

A strong interest in biomedical applications has led to PLA matrix composites with hydroxyapatite (HAP). These combine the osteoconductivity and bone-bonding ability of HAP with the absorbability and the easy processing of PLA and its copolymers. Jayabalan et al. described in their paper a composite of HAP NPs in a polymer matrix, with a compressive strength two times higher for the composite than for pure polymer. Their paper emphasizes that a combination of HAP NPs with the polymer matrix results from the modification of surface and calcination of HAP NPs. SEM images present 20 µm agglomerates of HAP NPs. The authors point to a large potential of the obtained material for bone fixation devices [26]. Calcium phosphate ceramics, such as HAP, are bioactive and similar to natural apatite found in bone tissue. Bone is a natural composite, consisting mainly of type I collagen, calcium phosphate, and water. Inorganic components account for 50–70% of the dry weight of bone, with the most important of them being crystalline nanohydroxyapatite [27,28,29,30,31]. Therefore, the composition of 1:1 by volume, proposed by the authors, has a potential for the production of osteoinductive materials with the required structure and engineering properties, which will be functional materials. Additional reinforcement comes from the use of stoichiometric HAP NPs. The nanostructure is preserved during processing.

Production of a material for a high-strength and bioresorbable implant is a complex problem. The composition of the composite is essential, but attention should also be drawn to its microstructure [24,25,26]. There are known methods for the preparation of composites with a high proportion of micrometric ceramics in the polymer matrix. Composites with a high ceramic phase content can be obtained by the infiltration of a ceramic matrix by a polymer, the mechanical grinding of components, or chemical methods (polymer dissolution and addition of ceramics) and extrusion [32,33,34,35,36,37,38]. Composite-forming methods can be axial or isostatic pressing. Russias et al. tested various proportions of a composite of polylactide and micrometric HAP particles. They proved that composites with the content of 70–80% of microHAP were characterized by mechanical properties corresponding to the human cortical bone. They also noticed that composites with such a composition were less homogeneous than those with less than 50% of HAP particles, i.e., more polymer. The SEM images presented in that publication disclose large HAP agglomerates. Russias et al. stated that the low polymer content in the composite and its non-uniform distribution did not achieve a high densification of the samples (for 80% of HAP they achieved the densification of 86%), which translated into unfavorable mechanical properties and degradation of the material [27]. It is a challenge to obtain a homogeneous material with good adhesion between the polymer and the ceramic. Such microstructures are a prerequisite for good mechanical properties. The greater the amount of ceramics, the more difficult the task. Gay et al. [38] describe the preparation of a composite of HAP NPs and PLLA by HAP NPs deagglomeration using wet attrition milling and further dispersion in chloroform. This procedure resulted in a homogeneous composite with an HAP NPs content of 25–50 wt%, which is characterized by a density of up to 97% and a maximum compressive strength of 100 MPa. Wolff et al. [24] describes obtaining a composite with 78 vol% of a ceramic using the spray granulation technology and uniaxial warm pressing. The obtained porosity is below 2 vol%. A previous article by Pietrzykowska et al. described methods of obtaining and forming the bioresorbable composite. The presented methods led to a composite with a preserved nanostructure and the compressive strength of 110 MPa. The presented work also showed problems with agglomeration of particles and their formation due to the amount of surface water in the nanopowder [39].

Moreover, composite filaments are created from phosphates and thermoplastic polymers. In their review paper, Fallon et al. underline the problem related to the increase in the quantity of the filler (i.e., ceramic particles) in thermosetting polymers per filament for 3D printing. They report that in line with the increase in the filler content, usually the decrease in homogeneity and dispersion of the composite, as well as the increase in viscosity are observed. This ultimately has an adverse impact on processability, the quality and the mechanical properties of the printed sample [40]. Dubinenko et al. describe a composite for 3D printing with the maximum HAP content of 50 wt%. The composite was obtained by dissolving PLA in chloroform, mixing with HAP in a ball mill, and subsequently after drying, it was processed in an extruder to obtain a filament for 3D printing. It was shown that the composite filament was characterized by a homogeneous structure. For 50%, a substantial increase of E to 8000 MPa was achieved, while pure PLLA has 2468 MPa [41]. 

Despite earlier studies on bioresorbable materials, there has been little research on composites with a bioactive ceramic matrix, combined with biodegradable polymers.

In this research, we focus on creating a composite characterized by a high degree of homogeneity, which can be formed from bioresorbable components. The base component of the composite is HAP NPs, which provides biological properties and strength (osteoinduction, Young’s modulus, and compressive strength) [29,42]. A polymer providing the implant with resistance to brittle cracking is the other component. Such a material could be used, for instance, for bone grafts. The application of such a biodegradable material in bone damage treatment reduces the risk of complications and the costs of treatment. The developed composite is characterized by a high homogeneity of the structure. A ceramic additive increases the strength of the thermoplastic polymer–polylactide (PLA) [43,44,45,46,47,48].

## 2. Materials and Methods

### 2.1. Materials Preparation

**Figure 1:**
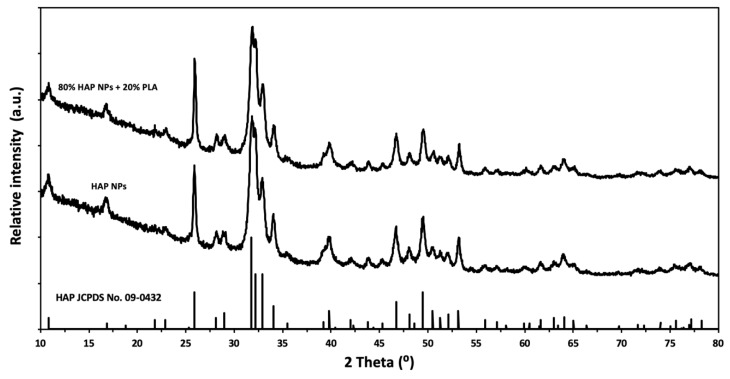
XRD of the HAP NPs and composite granules.

In this study, HAP NPs were prepared by the wet chemical precipitation method from calcium oxide (Fluka, Munich, Germany), orthophosphoric acid (Sigma-Aldrich, Steinheim, Germany) solution, and deionized water. The HAP NPs granules were obtained by the spray drying method using a spray dryer (Mini Spray Dryer b-290, Buchi, Flawil, Switzerland) with a fitted nozzle with the diameter of 1.4 mm. The inlet and outlet temperatures of the nozzle were adjusted to 220 °C, and 96 °C, respectively. Aqueous dispersions of HAP NPs, with the HAP NPs concentration of 10 wt%, were prepared using the homogenizing device. The specific surface area of HAP NPs was 77 m^2^/g and the density was 2.83 g/cm^3^. The Ca/P ratio was 1.73. The HAP average particle size calculated, based on the specific surface area and skeletal density result was 28 nm, and the average crystallite size was 29 nm. The average size of HAP NPs was equal to the average crystallite size, which means that the HAP NPs were built of single crystals. Figure 1 shows the results of the powder X-ray diffraction (XRD) measurements of the HAP NPs. 

PLA is a biodegradable and biocompatible synthetic polymer with the tensile yield strength of 62 MPa, tensile elongation of 3.5%, density of 1.24 g/cm^3^, and relative viscosity of 3.3. The polylactide used in this research is a commercial material with the trade name Ingeo biopolymer 3052D, produced by NatureWorks LLC, Minnetonka, MN, USA. The material takes the form of transparent granules, which are sized approximately 0.5 mm.

### 2.2. Materials Preparation

Composite granules (PLA/HAP NPs) of HAP NPs and PLA with a high content (up to 80 wt%) of calcium phosphate phase were prepared by a solvent evaporation method. First, the determined amounts of PLA were dissolved in dichloromethane to achieve a composite with the total organic fraction of 20 wt%. Next, HAP NPs granules were suspended in the polymer solution and subjected to magnetic stirring for two hours at room temperature. Subsequently, we conducted a high-pressure impregnation of PLA solution in HAP NPs granules. The suspensions were cast into Petri dishes. The dishes were left at room temperature for 12 h in order to allow full evaporation of the dichloromethane. A powder of the composite granules was prepared using ball-milling (FRITSCH Pulverisette 5, Weimar, Germany) and cryo-milling (IKA^®^ A11 basic analytical mill, Staufen, Germany).

### 2.3. Forming

**Figure 2:**
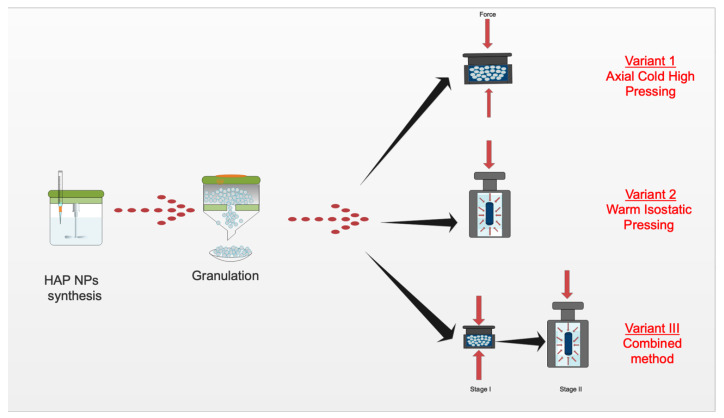
Diagram of the 80% HAP NPs + 20% PLA composite production process.

High-pressure forming was performed with three variants: (1) Cold high-pressure pressing, (2) warm isostatic pressing, and (3) a combination of the above two variants. Figure 2 shows the diagram of forming the composite. The LCP20 press in the Institute of High Pressure PAS was used during this research. The temperature was given and controlled by a proportional–integral–derivative controller (model RE93, Lumel S.A., Zielona Góra, Poland), together with a temperature sensor (T type thermocouple, model T-208p-K-1-300-40-2,5-SO-1-6-400, Termo-Precyzja, Wrocław, Poland).

Variant 1 involved cold pressing at a pressure of up to 1 GPa. The method involved filling a steel mold with the above-described granules and applying axial pressure at room temperature. Samples were kept under pressure for 10 s. The compaction density was investigated as a function of pressure. Five samples at 2 g each were pressed. A cuboid was formed with the dimensions of 4 × 4 × 35 mm. The mechanical strength was measured for three samples produced at a given pressure.

The second variant was warm isostatic pressing, with the consolidation temperature of up to 200 °C and the pressure of 75 MPa. Five samples at 2 g each of composite granules were placed in an elastic mold with the diameter of 8 mm and the height of 15 mm. These were pressed using isostatic pressure at 165 °C. The pressure vessel chamber was filled with methyl silicone oil. The optimum temperature was selected experimentally. 

Variant 3, which involved combining the above methods in two stages, used the following two conditions:

- first, axial pressing at 1 GPa
- then, warm isostatic consolidation at the temperature of 165 °C and 75 MPa.

The mechanical properties and densification were investigated.

### 2.4. Materials Characterization

The density measurements were performed with a helium pycnometer (model AccuPyc 1330, Micromeritics, Norcross, GA, USA) using an in-house procedure.

The specific surface area (SSA) of the powders was measured by the Brunauer-Emmett-Teller (BET) method (model Gemini 2360, V 2.01, Micromeritics, Norcross, GA, USA). The average diameter of the particles was calculated based on the specific surface area and density, assuming that all of the particles were spherical and identical [49]. 

Chemical composition of the powders: The chemical composition analysis of the powders was examined by inductively coupled plasma optical emission spectrometry (ICP-OES) with induction in argon plasma (Thermo Scientific, iCAP 6000 series, Cambridge, United Kingdom). The samples analyzed using ICP-OES were prepared as follows: 5 mg of powder was weighed in a 110 mL Teflon^®^ vessel, and 15 mL of deionized water (HLP 20 UV, Hydrolab, Straszyn, Poland) was added. Then, 6 mL of HNO_3_ was added, and the solution was subjected to one microwave heating cycle in the microwave reactor (Magnum II, Ertec, Wroclaw, Poland). After cooling, the sample volume was replenished to 50 mL with deionized water.

The densification of the material was investigated as a function of pressure. The densification and porosity of the consolidated material were checked at a pressure between 100 MPa and 1000 MPa. The compressions of HAP NPs and PLA/HAP NPs composite as a function of pressure were compared.

The thermogravimetry analysis (TG) was carried out using an STA 449 F1 Jupiter by Netzsch (Selb, Germany). The analysis was performed with a heating rate of 10 °C/min; the top temperature was 200 °C. 

The phase composition of the reaction products was analyzed by powder X-ray diffraction (Panalytical X’Pert PRO diffractometer, Cu Kα1, Panalytical, Almelo, The Netherlands). The patterns were collected at room temperature in the two-theta range 10–100° and with a step of 0.03°. The Scherrer equation determined the particle size.

SEM: The materials’ structure was examined by scanning electron microscopy (SEM) using the Ultra Plus microscope (ZEISS, Oberkochen, Germany). 

Uniaxial compression tests were carried out at room temperature using an MTS 858 (Eden Prairie, MN, USA) dynamic testing machine equipped with a ±15 kN transducer. The tests were conducted under the displacement control mode. The crosshead velocity was 20 μm/min. Strain was measured based on crosshead displacement. Samples with the length of 15 mm and the diameter of 8 mm were used for each test. Based on the load displacement data, the yield stress (YS) and ultimate compressive strength (UCS) were estimated.

Three-point bending tests were carried out at room temperature using an MTS QTest/10 (Eden Prairie, MN, USA) test universal testing machine equipped with a ±15 kN transducer. The tests were conducted under the displacement control mode. The crosshead velocity was 0.5 mm/min. Samples with the length of 20 mm and the cross-section of 4 mm × 4 mm were used for each test. The span was 12 mm. Based on the stress-strain curve, the ultimate bending strength was calculated.

The Vickers hardness measurement was performed using a hardness tester with a 100 g load.

Microcomputed tomography (micro-CT) was performed using SkyScan 1172 (Bruker, Kontich, Belgium), The X-ray source was 400 kV, 10 W, 5 µm pixel size.

## 3. Results and Discussion

**Figure 3:**
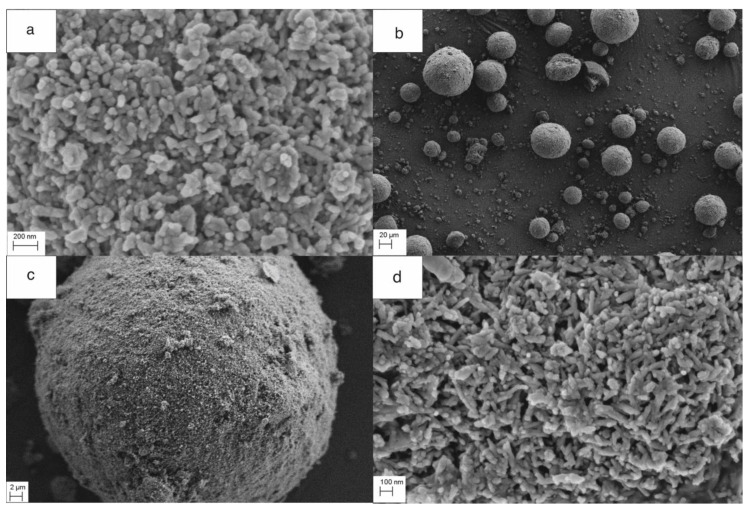
(**a**–**d**) The morphology of the HAP NPs and composite granules. (**a**) shows the structure in HAP NPs at the magnification of 100,000×, (**b**–**d**) composite granules of HAP NPs-PLA at the magnification of 500×, 5000× and 100,000×.

The average size of granules obtained using the spray drying and infiltration technology was 80 μm and their density was 2.26 g/cm^3^. The ratio of HAP NPs to PLA, as measured by TG, was 20% PLA and 80% HAP NPs by weight. The phase composition of the composite granules was investigated by XRD, which showed that the HAP NPs structure was preserved (Figure 1). The morphology of the granule samples was examined by SEM. The granules were round and homogeneous. PLA was well dispersed, and the boundary between the particle and the polymer was invisible (large areas of PLA were not observed). Figure 3a shows the size of the composite granules, and Figure 3b,c shows the spherical structure, density, nanoporosity, and size of HAP NPs. TG studies confirmed the proportion of HAP NPs and PLA as 80:20 by weight. The melting point of PLA was 175 °C. Nanoparticles tend to form agglomerates, which is the main obstacle in the formation of a homogeneous composite [50,51,52,53,54]. In our case, the obtained granulate is a controlled agglomerate of HAP NPs infiltrated with PLA. Figure 3c presents the achieved high homogeneity of the nanocomposite, at the same time preserving the structure and phase composition of HAP NPs.

**Figure 4:**
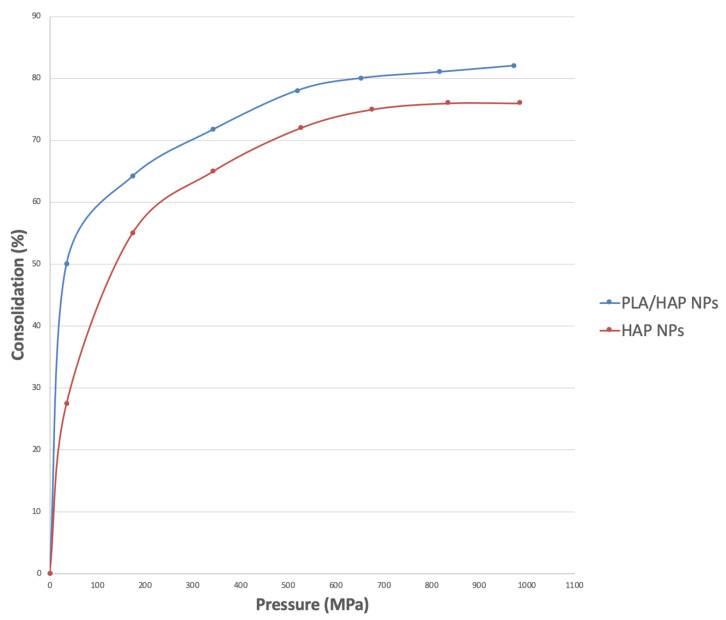
Densification of HAP NPs and composite granules (80 wt% of HAP NPs, 20 wt% of PLA) achieved during the uniaxial cold pressing.

The results of the forming using axial pressures of up to 1000 MPa (at room temperature) are as follows. Figure 4 shows a densification graph of PLA/HAP NPs composite granules. The materials were compared with pure HAP NPs as a function of pressure. The densification increased in line with the forming pressure. We see the fastest density increase in the range of up to 200 MPa. After that, the density increased slowly. Between 800 MPa and 1000 MPa, the densification was only a few percent. The densification of composite granules was higher than that for pure HAP NPs. For HAP NPs, we obtained a maximum densification of 82%, whereas for the composite granules it was 90%. An even better degree of densification was achieved for the composite granulate because round granules filled the steel mold more precisely, which is proven by the rapid increase in the densification (Figure 4). We are convinced that PLA in the composite fulfilled the role of a lubricant for HAP NPs, which ultimately enabled the achievement of a higher degree of densification. These results are similar to those achieved by Rakovsky et al. [34].

The results of compaction, using the warm isostatic technology, are as follows. The optimal compaction temperature for the composite of 80% HAP NPs and 20% PLA was 155 °C at a pressure of 75 MPa. The results of the warm isostatic technology at a temperature below the melting point (glass transformation: 60 °C at normal pressure) were checked. We managed to obtain solid moldings with a densification of 30% and a compressive strength of 100 MPa. When comparing this variant of forming with variant I, we noticed that, in order to achieve the densification of 70% in variant I (i.e., without temperature), the necessary pressure required is 300 MPa. In variant II, the applied temperature of 155 °C is the melting point of PLA, which contributes, together with pressure, to the viscosity of the polymer, thereby increasing the slip in the composite. Due to the high dispersion of the polymer and transition to the plastic state of PLA, the densification of 70% is achieved at 75 MPa and 165 °C.

After comparing the results of both variant, being axial pressure pressing and warm isostatic pressing, we selected and applied the best parameters for forming the composites in the third variant. The characteristic properties of compaction, compressive strength, and bending were compared. First, dense composite samples were obtained by axial pressing at 1000 MPa at room temperature. Then, the filled mold was placed in a steel chamber for isostatic pressing. The isostatic pressing process was carried out at the temperature of 155 °C and pressure of 75 MPa. The samples were kept in these conditions for 12 minutes. Then, they were removed from the vessel and cooled in air. The densification difference was observed after high-pressure densification and warm isostatic consolidation at 155 °C. During this experiment, the pressure was optimized for maximum densification of the composite material. The green body of the composites was condensed up to 85%.

**Figure 5:**
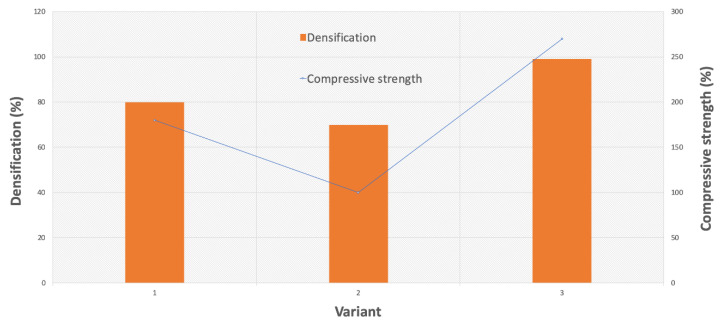
Comparison of three variants of forming, their impact on densification and porosity.

**Figure 6:**
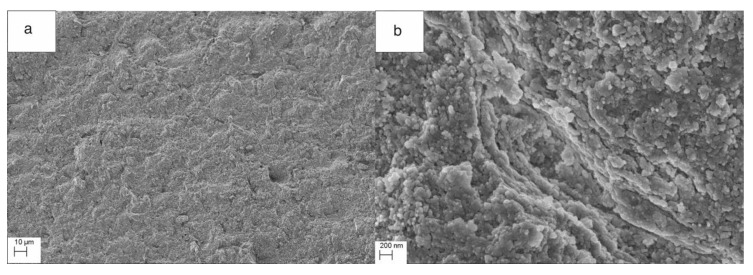
Fracture surface morphology of HAP NPs-PLA dense composites after a bending test for variant III, (**a**) Fracture at the magnification of 500×, (**b**) Composite structure at the magnification of 100,000×.

The densification, porosity, and mechanical properties were investigated. The results are shown in Table 1 and Figure 5. The highest densification displayed by the samples that were pressed using the two steps was 99%, whereas that displayed by the cold-pressed samples was only 80% (Figure 6). In Figure 5, we observed the increase in mechanical properties along with the rise in the degree of compaction. The best results were obtained for the third variant (the two-stage forming), in which the cold-pressed material at 1000 MPa was then compressed at 165 °C at a lower pressure. This process was carried out at the softening temperature of the polymer, which allowed the polymer to combine, and resulted in an increase in bending strength. The two-stage pressing was based on the maximum ceramic densification in the first step and on infiltration of the polymer in the second step. The combination method caused the removal of material stresses. It is not simple to remove the porosity when the densification degree is above 90% for ceramic nanocomposites [27]. The combination of two pressing methods permitted the application of variant I of the granulate, which improved mold bulk density and filling. In variant II, the polymer was used as a lubricant and adhesive.

Structural investigations showed a very homogeneous structure of the composites. The fracture surfaces of the samples after the bending tests showed that the structure of the composite was very homogeneous and brittle (Figure 6). Gay et al. [38] described that “At higher mineral contents, the rupture mechanism of the composite changes from ductile to brittle”. High-pressure pressing led to a high compaction of the composites. We assumed that the PLA polymer present in the composite granules acted as a lubricant during pressing. In the course of warm isostatic pressing, which took place at the softening temperature of the polymer, both compaction and infiltration occurred. We observed that the polymer in the ceramic matrix at the softening temperature combined. The effect was to increase the compressive and bending strength of the samples.

**Figure 7:**
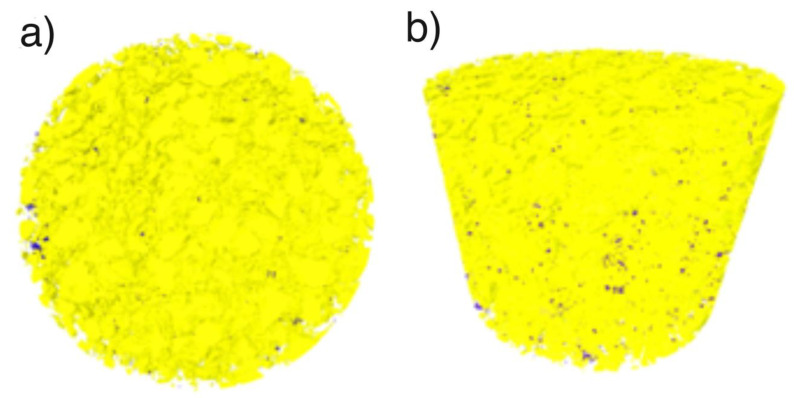
Three-dimensional reconstruction of HAP NPs (yellow) PLA (transparent) and pores (violet) using micro-CT, cylinder 4 mm height, 4 mm diameter: (**a**) Top view, (**b**) side view.

The micro-computed tomography studies of the composition and porosity of the composite formed by the third variant showed the porosity of up to 1 vol%. The PLA volume fraction was 21%. A three-dimensional reconstruction showed that the structure was homogeneous. Figure 7a shows a top view of the sample: Violet color pores are disclosed against a yellow background, their share is 1% of the volume. The micro tomography tests did not disclose a two-phase nature of the obtained composite. At the resolution level of 5 μm of the device, the material is homogeneous. Figure 7b is a side view and consequently it shows a distribution of porosity and homogeneity of the material in the cross-section.

The mechanical properties of the composites were compared with the mechanical properties of polylactide and the sintered ceramic body with HAP NPs from the literature. The highest compressive strength for a sintered HAP NPs composite was 208.6 MPa [53]. Dan Wu et al. obtained compressive strength below 80 MPa for PLA and its composites [54]. The result is unique in terms of the achieved high homogeneity of HAP NPs and of the polymer in the obtained granulate. Ultimately, a solid sample, with a hybrid structure and good mechanical properties, was obtained.

As the bending strength did not change, we did not observe changes related to the blocking of cracks as we did with a decrease in porosity. As widely known, the decrease in porosity and internal stresses increase the strength. We monitored the hardness of the formed composites. The cold-pressed samples had the highest repeatability of results. The warm-pressed samples, probably because of porosity, had the smallest hardness HV1. The mixed method allowed us to obtain materials with a good repeatability of results, and HV1 was 52 ± 5.

In summary, the two-step method allowed us to achieve very high densification and mechanical properties. This was possible because a tight bonding between PLA and HAP NPs was obtained within the pores of the infiltrated granules, and because the granules presented excellent properties for the isostatic pressing technology. 

A highly dense and homogeneous composite was formed as a result of the use of pressing technologies. The results are promising because a composite, characterized by a high dispersion of nanoparticles, was obtained, at the same time preserving their phase structure. The next step should include a degradation test with in vitro conditions. The composites obtained by our method have stronger mechanical properties than similar materials previously discussed in the literature. In the literature, Wolff obtained a composite with 78 vol% of a ceramic, using the spray granulation technology and uniaxial warm pressing. The obtained porosity was below 2 vol% and the mechanical strength was 100 MP [39]. These results prove a novelty: The achievement of a highly homogeneous material with a high quantity of ceramic particles. The high degree of homogeneity of the composite granules contributed substantially to the compressibility of the material and its final properties.

## 4. Conclusions

The results of this paper constitute a response to the lack of sufficient mechanical strength of the bioresorbable composites being hydroxyapatite nanoparticles in a polymer matrix (e.g., polylactic acid (PLA)) for bone substitution purposes in bone regeneration, which have been obtained so far. The aim and novelty of the research was to obtain a homogeneous hybrid composite, characterized by a high degree of densification, with the HAP NPs content of up to 80 wt%, without the presence of agglomerates of NPs.

We have developed a two-step method for a bioresorbable composite with a very high content of a ceramic in a polymer matrix. The composite has potential for orthopedic applications as it achieved the compressive strength of 375 MPa, the Young’s modulus of 7 GPa, and the densification of 99%. The first step was to prepare composite granules of porous HAP NPs granules and PLA by high-pressure infiltration. The second step was the consolidation of the composite granules. 

Three consolidation variants for the forming were tested. The best results were obtained by compaction in the third variant: uniaxial cold pressing and subsequent isostatic pressing at 155 °C. We successfully formed a composite, consisting of HAP NPs at 80 wt% and a bioresorbable polymer (PLA) for the balance. The composite had a homogeneous structure, and we observed good adhesion between the polymer and the ceramic. The obtained composite had an average HAP NPs particle size of 28 nm. This approach permitted us to consolidate the composite without nanoparticle growth and degradation of the polymer.

The technology can be used to form a thermosetting composite with a high content of a ceramic phase at a relatively low temperature of up to 200 °C. The manufactured composites are promising materials for applications such as implants in bone regeneration. 

## Figures and Tables

**Table 1:** Mechanical properties of the 80% HAP NPs + 20% PLA composite for different variants.

Parameter	Variant 1 (Cold Pressing)	Variant 2 (Warm Pressing)	Variant 3 (Cold + Warm Pressing)
**Compressive strength (MPa)**	180 ± 7	100 ± 6	370 ± 7
**Bending strength (MPa)**	19 ± 5	19 ± 4	23 ± 4
**Porosity (%)**	20	30	1

## References

[1] Shi D. (2005). Introduction to Biomaterials.

[2] Ramakrishna S. (2010). Biomaterials: A Nano Approach.

[3] Matsumoto M., Chosa E., Nabeshima K., Shikinami Y., Tajima N. (2002). Influence of bioresorbable, unsintered hydroxyapatite/poly-L-lactide composite films on spinal cord, nerve roots, and epidural space. J. Biomed. Mater. Res..

[4] Dziadeka M., Stodolak-Zych E., Cholewa-Kowalska K. (2017). Biodegradable ceramic-polymer composites for biomedical applications: A review. Mater. Sci. Eng. C.

[5] Yaszemski M.J. (2003). Biomaterials in Orthopedics.

[6] Godavitarne C., Robertson A., Peters J., Rogers B. (2017). Biodegradable materials. Orthop. Trauma.

[7] Gallo A., Agnese V., Coronnello C., Raffa G.M., Bellavia D., Conaldi P.G., Pilato M., Pasta S. (2018). On the prospect of serum exosomal miRNA profiling and protein biomarkers for the diagnosis of ascending aortic dilatation in patients with bicuspid and tricuspid aortic valve. Int. J. Cardiol..

[8] Pasta S., Gentile G., Raffa G.M., Bellavia D., Chiarello G., Liotta R., Luca A., Scardulla C., Pilato M. (2017). In Silico Shear and Intramural Stresses Are Linked to Aortic Valve Morphology in Dilated Ascending Aorta. Eur. J. Vasc. Endovasc. Surg..

[9] Modjarrad K., Ebnesajjad S. (2014). Handbook of Polymer Applications in Medicine and Medical Devices.

[10] Tsuji H. (2016). Poly(lactic acid) stereocomplexes: A decade of progress. Adv. Drug Deliv. Rev..

[11] Tschakaloff A., Losken H.W., Lalikos J., Link J., Mooney M.P., von Oepen R., Michaeli W., Losken A. (1993). Experimental studies of DL-polylactic acid biodegradable plates and screws in rabbits: Computed tomography and molecular weight loss. J. Craniofac. Surg..

[12] Farah S., Anderson D.G., Langer R. (2016). Physical and mechanical properties of PLA, and their functions in widespread applications—A comprehensive review. Adv. Drug Deliv. Rev..

[13] Gleadall A., Pan J., Kruft M.A., Kellomȧki M. (2014). Degradation mechanisms of bioresorbable polyesters. Part 1. Effects of random scission, end scission and autocatalysis. Acta Biomater..

[14] Middleton J., Tipton A.J. (2000). Synthetic biodegradable polymers as orthopedic devices. Biomaterials.

[15] Zhou H., Lawrence J.G., Bhaduri S.B. (2012). Fabrication aspects of PLA-CaP/PLGA-CaP composites for orthopedic applications: A review. Acta Biomater..

[16] Petisco-Ferrero S., Pérez Álvarez L., Ruiz-Rubio L., Vilas Vilela J.L., Sarasua J.R. (2018). Plasma poly(acrylic acid) compatibilized hydroxyapatite-polylactide biocomposites for their use as body-absorbable osteosynthesis devices. Compos. Sci. Technol..

[17] Rakovsky A., Gotman I., Rabkin E., Gutmanas E.Y. (2014). β-TCP–polylactide composite scaffolds with high strength and enhanced permeability prepared by a modified salt leaching method. J. Mech. Behav. Biomed. Mater..

[18] Agrawal C.M., Athanasiou K.A. (1997). Technique to control pH in vicinity of biodegrading PLA–PGA implants. J. Biomed. Mater. Res..

[19] Cordewener F.W., Schmitz J.P. (2000). The future of biodegradable osteosyntheses. Tissue Eng..

[20] Kashirina A., Yao Y., Liu Y., Leng J. (2019). Biopolymers for bone substitutes: A review. Biomater. Sci..

[21] Tajbakhsh S., Hajiali F. (2017). A comprehensive study on the fabrication and properties of biocomposites of poly(lactic acid)/ceramics for bone tissue engineering. Mater. Sci. Eng. C.

[22] Zagho M.M., Hussein E.A., Elzatahry A.A. (2018). Recent Overviews in Functional Polymer Composites for Biomedical Applications. Polymers.

[23] Boczkowska A., Konopka K., Kurzydłowski K.J. (2006). Effect of elastomer structure on ceramic–elastomer composite properties. J. Mater. Process. Technol..

[24] Wolff F.H., Salikova V., Antonyuka S., Heinricha S., Schneiderb G.A. (2014). Novel, highly-filled ceramic–polymer composites synthesized by a spouted bed spray granulation process. Compos. Sci. Technol..

[25] Konopka K., Boczkowska A., Batorski K., Szafran M., Kurzydłowski K.J. (2004). Microstructure and properties of novel ceramic–polymer composites. Mater. Lett..

[26] Jayabalan M., Shalumon K.T., Mitha M.K., Ganesan K., Epple M. (2010). Effect of hydroxyapatite on the biodegradation and biomechanical stability of polyester nanocomposites for orthopaedic applications. Acta Biomater..

[27] Russias J., Saiz E., Nalla R.K., Gryn K., Ritchie R.O., Tomsia A.P. (2006). Fabrication and mechanical properties of PLA/HA composites: A study of in vitro degradation. Mater. Sci. Eng. C.

[28] Ramakrishna S., Mayer J., Wintermantel E., Leong K.W. (2001). Biomedical applications of polymer-composite materials: A review. Compos. Sci. Technol..

[29] Kuśnieruk S., Wojnarowicz J., Chodara A., Chudoba T., Gierlotka S., Lojkowski W. (2016). Influence of hydrothermal synthesis parameters on the properties of hydroxyapatite nanoparticles. Beilstein J. Nanotechnol..

[30] Takikawa K., Akao M. (1996). Fabrication of transparent hydroxyapatite and application to bone marrow derived cell/hydroxyapatite interaction observation In-Vivo. J. Mater. Sci. Mater. Med..

[31] Wilberforce S.I.J., Finlayson C.E., Bes S.M., Cameron R.E. (2011). The influence of the compounding process and testing con- ditions on the compressive mechanical properties of poly(D,L- lactide-co-glycolide)/a-tricalcium phosphate nanocomposites. J. Mech. Behav. Biomed. Mater..

[32] Ignjatovic N., Uskokovic D. (2004). Synthesis and application of hydroxyapatite/polylactide composite biomaterial. Appl. Surf. Sci..

[33] Ignjatovic N., Ignjatovic N., Suljovrujic E., Budinski-Simendic J., Krakovsky I., Uskokovi D. (2004). Evaluation of hot-pressed hydroxyapatite/poly-L-lactide composite biomaterial characteristics. J. Biomed. Mater. Res. Part B Appl. Biomater..

[34] Rakovsky A., Gutmanas E.Y., Gotman I. (2010). Ca-deficient hydroxyapatite/polylactide nanocomposites with chemically modified interfaces by high pressure consolidation at room temperature. J. Mater. Sci..

[35] Delabarde C., Plummer C.J.G., Bourban E., Manson J.A.E. (2010). Solidification behavior of PLLA/nHA nanocomposites. Compos. Sci. Technol..

[36] Wagoner F.A.J. (2011). A review of the mechanical behavior of CaP and CaP/polymer composites for applications in bone replacement and repair. Acta Biomater..

[37] Mathieu L.M., Bourban P.E., Månson J.A.E. (2006). Processing of homogeneous ceramic/polymer blends for bioresorbable composites. Compos. Sci. Technol..

[38] Gay S., Arostegui S., Lemaitre J. (2009). Preparation and characterization of dense nanohydroxyapatite/PLLA composites. Mater. Sci. Eng. C.

[39] Pietrzykowska E., Mukhovskyi R., Chodara A., Wojnarowicz J., Koltsov I., Chudoba T., Łojkowski W. (2019). Composites of polylactide and nano-hydroxyapatite created by cryomilling and warm isostatic pressing for bone implants applications. Mater. Lett..

[40] Fallon J.J., McKnight S.M., Bortner M.J. (2019). Highly loaded fiber filled polymers for material extrusion: A review of current understanding. Addit. Manuf..

[41] Dubinenko G.E., Zinoviev A.L., Bolbasov E.N., Novikov W.T., Tverdokhlebov S.I. (2020). Preparation of Poly(L-lactic acid)/Hydroxyapatite composite scaffolds by fused deposit modeling 3D printing. Mater. Today Proc..

[42] Azevedo M.C., Reis R.L., Claase M.B., Grijpma D.W., Feijen J. (2003). Development and properties of polycaprolactone/hydroxyapatite composite biomaterials. J. Mater. Sci. Mater. Med..

[43] Neumann M., Epple M. (2006). Composites of calcium phosphate and polymers as bone substitution materials. Eur. J. Trauma.

[44] Ignjatovic N., Savic V., Najman S., Plavsic M., Uskokovic D. (2001). A study of HAp/PLLA composite as a substitute for bone powder, using FT-IR spectroscopy. Biomaterials.

[45] Hong Z., Zhang P., He C., Qiu X., Liu A., Chen L., Chen X., Jing X. (2005). Nano-composite of poly(l-lactide) and surface grafted hydroxyapatite: Mechanical properties and biocompatibility. Biomaterials.

[46] Ma F., Chen S., Liu P., Geng F., Li W., Liu X., He D., Pan D. (2016). Improvement of β-TCP/PLLA biodegradable material by surface modification with stearic acid. Mater. Sci. Eng. C.

[47] Rakovsky A., Gotman I., Rabkin E., Gutmanas E.Y. (2013). Strong bioresorbable Ca phosphate–PLA nanocomposites with uniform phase distribution by attrition milling and high pressure consolidation. J. Mech. Behav. Biomed. Mater..

[48] Neuendorf R.E., Saiz E., Tomsia A.P., Ritchie R.O. (2008). Adhesion between biodegradable polymers and hydroxyapatite: Relevance to synthetic bone-like materials and tissue engineering scaffolds. Acta Biomater..

[49] Wojnarowicz J., Opalinska A., Chudoba T., Gierlotka S., Mukhovskyi R., Pietrzykowska E., Sobczak K., Lojkowski W. (2016). Effect of water content in ethylene glycol solvent on the size of ZnO nanoparticles prepared using microwave solvothermal synthesis. J. Nanomater..

[50] Cierech M., Wojnarowicz J., Szmigiel D., Bączkowski B., Grudniak A., Wolska K., Łojkowski W., Mierzwińska-Nastalska E. (2016). Preparation and characterization of ZnO-PMMA resin nanocomposites for denture bases. Acta Bioeng. Biomech..

[51] De Luna M.S., Galizia M., Wojnarowicz J., Rosa R., Lojkowski W., Leonelli C., Acierno D., Filippone G. (2014). Dispersing hydrophilic nanoparticles in hydrophobic polymers: HDPE/ZnO nanocomposites by a novel template-based approach. eXPRESS Polym. Lett..

[52] Fu S., Sun Z., Huang P., Li Y., Hu N. (2019). Some basic aspects of polymer nanocomposites: A critical review. Nano Mater. Sci..

[53] Rid A., Firdaus R., Mulyadi I.H., Affi J. (2020). Enhancing the physical and mechanical properties of pellet-shaped hydroxyapatite by controlling micron- and nano-sized powder ratios. Ceram. Int..

[54] Wu D., Spanou A., Diez-Escudero A., Persson C. (2020). 3D-printed PLA/HA composite structures as synthetic trabecular bone: A feasibility study using fused deposition modeling. J. Mech. Behav. Biomed. Mater..

